# The use of embryonic chicken eggs as an alternative model to evaluate the virulence of *Salmonella enterica* serovar Gallinarum

**DOI:** 10.1371/journal.pone.0238630

**Published:** 2020-09-10

**Authors:** Jun-feng Zhang, Bai Wei, Se-Yeoun Cha, Ke Shang, Hyung-Kwan Jang, Min Kang

**Affiliations:** 1 Department of Veterinary Infectious Diseases and Avian Diseases, College of Veterinary Medicine and Center for Poultry Diseases Control, Jeonbuk National University, Iksan, South Korea; 2 Bio Disease Control(BIOD) Co., Ltd., Iksan, Republic of Korea; Panstwowy Instytut Weterynaryjny - Panstwowy Instytut Badawczy w Pulawach, POLAND

## Abstract

*Salmonella enterica* serovar Gallinarum (*S*. Gallinarum) can cause fowl typhoid, a severe systemic disease responsible for considerable economic losses. Chicken pathogenicity test is the traditional method for assessing the virulence of *S*. Gallinarum. However, this method is limited by several factors, including ethical considerations, costs, and the need for specialized facilities. Hence, we established a chicken embryo lethality assay (ELA) model to determine the virulence of *S*. Gallinarum. Three virulent and three avirulent representative strains, which were confirmed by the chicken pathogenicity test, were used to perform the ELA. The most significant difference between the virulent and avirulent strains could be observed when 13-day-old embryos were inoculated via the AC route and incubated for 5 days. Based on a 50% embryo lethal dose (ELD_50_), isolates considered to be virulent had a Log_10_ELD_50_ of ≤ 4.0, moderately virulent strains had a Log_10_ELD_50_ of 4.0−6.1, and avirulent isolates had a Log_10_ELD_50_ of ≥ 6.1. Different abilities to invade the liver of embryos were found between the virulent and avirulent strains by a growth curve experiment *in vitro*. The maximum colony-forming units (CFU) of the virulent strain was about 10,000 times higher than that of the avirulent strain in the liver at 5 days post infection. The ELA results of 42 field strains showed that thirty-two strains (76.2%) were virulent, nine were moderately virulent (21.4%), and one strain was avirulent (2.4%). In conclusion, these results suggest that the ELA can be used as an alternative method to assess the virulence of *S*. Gallinarum, which will contribute to the study of virulence genes, virulence evolution, pathogenic mechanisms and vaccine development.

## Introduction

*Salmonella enterica* serovar Gallinarum (*S*. Gallinarum) is the causative agent of fowl typhoid (FT), a serious systemic disease that causes huge economic losses to the commercial poultry industry [1]. This disease occurs at all ages in chickens and is characterized by severe anorexia, weight loss, depression, diarrhea, decreased egg production, and high morbidity and mortality [2]. FT has almost been eradicated from developed countries, such as Australia, North America and most European countries. However, in many developing countries such as Africa, Asia, the Middle East and Central and South America, it is still an important poultry disease with significant economic impact [3]. In South Korea, since the first FT outbreak was reported in 1992, the disease has occurred nationwide, with most cases occurring in the brown egg layers, which constitute the majority of the commercial egg industry [4].

Many strategies have been adopted to reduce the occurrence of FT, including the establishment of hygiene standards and the use of antibiotics and vaccines [5]. Among them, vaccination has been found to be the most practical and effective strategy for controlling FT [3]. Currently, most studies are focused on the development of vaccines against FT [6]. To develop a vaccine, animal models are often used for safety and effectiveness evaluation. For example, the chicken pathogenicity test is the most commonly used infection model in FT. However, unnecessary animal experiments should be avoided and their ethical aspects should be considered. Various acts and laws have been passed to control the unethical use of animals and minimize the suffering of animals during experiments [7]. Even if animals are used for the final evaluation, it is necessary that alternative methods are applied during the developmental stages. In addition, the cost of animal experiments is high, requiring specialized facilities and specially trained personal, which further limits the application of such infection tests [8].

In order to overcome the shortcomings related to animal experiments and to avoid unethical procedures, various animal experiment alternatives have been proposed, such as computer models, cell and tissue cultures, and alternative organisms [7].

The chicken embryo lethality assay (ELA) can be used as an alternative tool to study the virulence of various pathogens including bacteria (*Escherichia coli*, *Francisella* spp., *Staphylococcus aureus*, *Clostridium perfringens*, *Yersinia enterocolítica*, *Campylobacter jejuni*, *Riemerella anatipestifer*, *Listeria monocytogenes*, *Enterococcus faecalis*), fungi (*Candida albicans*, *Aspergillus fumigatus*), parasites (*Eimeria tenella*) and viruses (West Nile virus, Japanese encephalitis virus) [9–20]. It has advantages such as being a faster, more sensitive, cheaper, more specific and relatively simple assay without any ethical considerations.

In the case of *Salmonella*, the reports of the use of ELA mainly involve *Salmonella enterica* serovar Pullorum (*S*. Pullorum) and *Salmonella enterica* serovar Typhimurium (*S*. Typhimurium) [21–23]. However, to our knowledge, an ELA of *S*. Gallinarum has not been previously reported. *S*. Gallinarum is a host-adapted serovar, and can be transmitted to chicks through eggs, which suggests that it can replicate and multiply in chicken embryos. Therefore, we speculated that ELA can be used as an alternative to assess the virulence of *S*. Gallinarum. Each pathogen has its own characteristics, and it is important to study the corresponding pathogenicity criteria. Moreover, virulence evaluation of *S*. Gallinarum isolates contributes to the study of virulence genes, virulence evolution and pathogenic mechanisms, as well as vaccine development. In this study, we established a chicken embryo model for evaluating the virulence of *S*. Gallinarum strains and applied this model to field strains from clinical cases of FT.

## Materials and methods

### Ethical statement

All experimental and animal management procedures were undertaken in accordance with the requirements of the Animal Care and Ethics Committee of Jeonbuk National University. The animal facility at Jeonbuk National University is fully accredited by the National Association of Laboratory Animal Care (approval number: CBNU 2020–059).

### Bacterial strains and growth conditions

Strains of *S*. Gallinarum 287/91 (NCTC 13346), A17-DW-005, and A18-GCVP-014 (field strains used in the present study) were selected as representative virulent strains and the vaccine Nobilis SG9R (Intervet International, Boxmeer, The Netherlands), A17-DW-005Δ*spiC* (*spiC* is a virulence factor encoded within the *Salmonella* pathogenicity island 2) and A17-DW-005Δ*waaJ*Δ*spiC* were selected as representative avirulent strains [24, 25]. The above mutant strains were constructed in our lab. Forty-two field strains were isolated from clinical samples of chickens (layer, broiler, and Korean native chicken) at necropsy from 2016 to 2019 in South Korea. All strains were stored in Luria-Bertani (LB) medium containing 20% glycerol at −70°C, and then cultured on LB agar at 37°C for 24 h. Before each experiment, a single colony was picked and inoculated in LB broth and incubated overnight at 37°C with a shaking speed of 180 rpm, and the culture broth was then used for the assay.

### Effect of chicken embryo inoculation age and route on virulence

Specific pathogen-free (SPF) chicken embryos (Hy-Vac Laboratory, Redfield, Iowa, USA) were incubated until use in a constant temperature incubator at 37°C with 40 to 60% humidity. To determine the optimal inoculation age, 6, 10, 13, and 16-day-old embryos were selected. To determine the optimal inoculation route, allantoic cavity (AC), chorioallantoic membrane (CAM) and yolk sac (YS) were selected. Six-day-old embryos were inoculated only via the YS. Ten-day-old, 13-day-old, and 16-day-old embryos were inoculated via the AC and CAM. Three virulent strains 287/91, A17-DW-005, A18-GCVP-014 and one avirulent strain SG9R were used in this experiment.

Bacteria were grown to a density of 1.1 by OD_590_ (approximately 1×10^9^ CFU/ml) and different doses were obtained by serial 10-fold dilution. Fifteen chicken embryos were used per dilution. Vitality was assessed every 12 h by candling for 5 days and deaths were recorded. Embryos that died within 24 h of inoculation were assumed to have suffered lethal trauma during the inoculation and were removed from the experiment. Different doses were used to determine the 50% embryo lethal dose (ELD_50_) of each strain [26]. Dead embryos and embryos that survived the experiment were chilled for at least 4 h at 4°C followed by necropsy. The following scoring systems were used to assess the gross lesions of the embryonic body and liver. Embryonic body: 1 for normal, 2 for medium body size, 3 for small body size and 4 for small body size plus hemorrhage. Embryonic liver: 1 for normal, 2 for swelling, 3 for few necrotic foci, 4 for multiple necrotic foci. Bacterial re-isolation was performed from the AC fluid and the embryonic liver.

### Chicken pathogenicity test

The eleven selected *S*. Gallinarum strains were used to challenge chickens to confirm their virulence. Ten *Salmonella*-free 4-day-old Hy-Line brown layers in each group were used to evaluate the virulence of the *S*. Gallinarum strains *in vivo*. In order to guarantee the best environmental conditions, the isolator conditions (temperature, humidity, ventilation) were constantly monitored. The whole staff taking care of or handling the laboratory animals was well trained. Each of the *S*. Gallinarum strains was orally inoculated into the chickens at a dose of 10^8^ colony-forming units (CFU) [27]. After inoculation, the chickens were checked twice a day for 14 days. The clinical signs were scored as 0 for being normal, 1 for being depression and ruffled feathers, 2 for depression, ruffled feathers, respiratory distress and 3 for the above mentioned clinical signs plus anorexia, emaciation and green-yellowish diarrhea, and 4 for death. When birds showed the clinical score of 3 (humane endpoint), the chickens were humanely sacrificed by cervical dislocation performed by trained veterinarians immediately [28]. Despite our efforts, some of the chickens used in the present study died before euthanasia (natural death by fowl typhoid). All dead animals including natural death and euthanasia were necropsied in order to evaluate the presence of *S*. Gallinarum. Surviving chickens were euthanized at 14 days post-inoculation (dpi), and bacterial re-isolation was conducted.

### *In ovo* growth curve

To compare the growth curves of virulent strains and avirulent strains *in ovo*, embryos were inoculated with three virulent strains 287/91, A17-DW-005, A18-MRA-014, and one avirulent strain SG9R. Twenty 13-day-old eggs inoculated with 10^4^ CFU per strain via the AC were sacrificed at 0, 3, 6, 12, 18, and 24 h and daily thereafter for 4 days (a total of 5 days). Three eggs were used for each time point. A group inoculated with sterile PBS was also assessed. The AC fluids and livers were collected aseptically. The viable bacterial counts were estimated by plating dilutions of the AC fluid and homogenized liver onto MacConkey agar plates.

### Virulence assessment of the field strains by ELA

To evaluate the virulence of field strains, 42 field strains that originated from clinical cases were tested. ELD_50_ values were determined by inoculating different dilutions of the strains into the AC of 13-day-old chicken embryos according to the method described above. Fifteen eggs were inoculated per dilution.

### Statistical analyses

Statistical analysis was performed using SPSS version 21.0 (SPSS Inc., Chicago, IL, USA). ELD_50_ values and 95% confidence intervals (CIs) were calculated by using probit analysis of Bliss [29]. The one-way analysis of variance (ANOVA) was used for the analysis of significant differences between ELD_50_ values of virulent and avirulent strains using different age/route combinations. One-way ANOVA was also used for the analysis of significant differences in the CFU of different strains at the indicated time points from the AC fluid and embryonic liver, respectively. Differences were considered statistically significant at: * *P* <0.05, ** *P* <0.01, *** *P* <0.001.

## Results

### Effect of chicken embryo inoculation age and route on virulence

To determine the optimal inoculation age and route, different embryo ages and inoculation routes were selected. As shown in Table 1, in terms of the age of the chicken embryo, ELD_50_ values obtained from 13-day-old chicken embryos were higher than those obtained from 10-day-old chicken embryos. Similarly, ELD_50_ values obtained from 16-day-old chicken embryos were higher than those obtained from 13-day-old chicken embryos.

**Table 1:** Effect of the age of the chick embryo on the virulence by different inoculation routes.

Virulence	Strain	Route	Log_10_ELD_50_ (95% CI)^a^			
			6 d^b^	10 d	13 d	16 d
Virulent	287/91	AC	Nd^c^	<1	2.7 (2.1−3.1)***	3.4 (2.9−3.8)***
	A17-DW-005		Nd	<1	2.2 (1.4−2.7)***	3.1 (2.6−3.6)***
	A18-GCVP-014		Nd	<1	3.5 (2.9−4.0)***	3.9 (3.1−4.6)***
Avirulent	SG9R		Nd	2.3 (1.8−2.8)	6.5 (6.1−6.9)	6.5 (6.0−7.0)
Virulent	287/91	CAM	Nd	<1	2.1 (1.6−2.6)***	4.2 (3.5−4.7)***
	A17-DW-005		Nd	<1	1.9 (0.9−2.4)***	4.1 (3.6−4.5)***
	A18-GCVP-014		Nd	<1	2.8 (2.4−3.2)**	4.4 (3.9−5.1)**
Avirulent	SG9R		Nd	2.4 (1.6−2.7)	3.9 (3.4−4.5)	5.4 (4.9−6.1)
Virulent	287/91	YS	<1	Nd	Nd	Nd
	A17-DW-005		<1	Nd	Nd	Nd
	A18-GCVP-014		<1	Nd	Nd	Nd
Avirulent	SG9R		<1	Nd	Nd	Nd

^a^95% CI: 95% confidence interval.

^b^Age of chicken embryo (days).

^c^Nd: not done.

**P < 0.01,

***P < 0.001 (vs. SG9R).

Regarding the inoculation route, all 6-day-old chicken embryos died when inoculated by YS, which indicated that virulent and avirulent strains could not be distinguished through the YS route. Based on the results of statistical analysis, 13 day/AC (*P* = 0.000001), 13 day/CAM (*P* = 0.001371), 16 day/AC (*P* = 0.000004), and 16 day/CAM (*P* = 0.006511) (S2 Table) could be used to distinguish between virulent and avirulent strains. When the 13-day-old chicken embryos were inoculated by the AC, the most significant difference between the ELD_50_ values of the virulent and avirulent strains was manifested. Therefore, 13 day/AC was the optimal inoculation combination through which the virulent and avirulent strains could be distinguished to the greatest extent.

### Chicken pathogenicity test

Strains 287/91, A17-DW-005, and A18-GCVP-014 showed 100% mortality over 14 days (*n* = 10) (Table 2). A16-MRA-029, A18-MRA-014, A19-DW-008, A19-DW-013, and A17-DW-005Δ*waaJ* showed 50−70% mortality and 50−80% of chickens showed clinical signs at 14 dpi. Clinical signs and mortality were not observed for SG9R, A17-DW-005Δ*spiC*, or A17-DW-005Δ*waaJ*Δ*spiC*. In addition, it should be noted that six chickens died before reaching the humane endpoint, and the other 110 chickens were euthanized. After necropsy, all bacterial re-isolation results were *Salmonella* positive.

**Table 2:** Experimental infection of 4-day-old Hy-Line brown layers with *S*. Gallinarum strains with different virulences.

Strain	Number of chickens found dead at indicated dpi^b^														Accumulated chicken mortality (%)^c^	Number with clinical signs at 14 dpi (%)^a^	Rate of re-isolation (*n* = 10)^d^	Log_10_ELD_50_	95% CI
	Day 1	Day 2	Day 3	Day 4	Day 5	Day 6	Day 7	Day 8	Day 9	Day 10	Day 11	Day 12	Day 13	Day 14					
287/91			1		1			3	2	1	1		1		10/10 (100)	10/10 (100)	10/10 (100)	2.7	2.1−3.1
A17-DW-005			1	4	3	2									10/10 (100)	10/10 (100)	10/10 (100)	2.2	1.4−2.7
A18-GCVP-014			1	2	3	3		1							10/10 (100)	10/10 (100)	10/10 (100)	3.5	2.9−4.0
A16-MRA-029						1	2	3							6/10 (60)	7/10 (70)	8/10 (80)	5.1	4.5−5.6
A18-MRA-014			1	1	1		1		1			1	1		7/10 (70)	8/10 (80)	7/10 (70)	5.0	4.5−5.7
A19-DW-008				5					1						6/10 (60)	6/10 (60)	9/10 (90)	4.1	3.6−4.6
A19-DW-013			2				1				1	1	2		7/10 (70)	7/10 (70)	10/10 (100)	4.1	3.6−4.5
A17-DW-005ΔwaaJ ^e^				1	1				1						3/6 (50)	3/6 (50)	3/6 (50)	5.6	5.0−6.2
SG9R															0/10 (0)	0/10 (0)	6/10 (60)	6.5	6.1−6.9
A17-DW-005ΔspiC															0/10 (0)	0/10 (0)	10/10 (100)	6.7	6.1−7.5
A17-DW-005ΔwaaJΔspiC															0/10 (0)	0/10 (0)	2/10 (20)	7.8	7.1−9.0
PBS															0/10 (0)	0/10 (0)	0/10 (0)	NA^f^	NA

^a^Number of chickens showing clinical signs/total number of inoculated chickens.

^b^Dpi: day post infection.

^c^Number of dead chickens/total number of inoculated chickens.

^d^Re-isolation was done on 14 dpi.

^e^7-day-old Hy-Line brown layers were intramuscularly inoculated with A17-DW-005Δ*waaJ*.

^f^NA: Not available.

### Determination of virulence of representative strains by ELA

As shown in Table 2, the Log_10_ELD_50_ values of the virulent strains were 4.0 or less (4.0 was the highest value within the 95% CIs among the virulent strains), whereas the Log_10_ELD_50_ values of the avirulent strains were 6.1 or greater (6.1 was the lowest value within the 95% CIs among the avirulent strains). Therefore, the Log_10_ELD_50_ value range of the moderately virulent strains could be defined as 4.0−6.1. None of the embryos inoculated with PBS died during the 5 days.

Based on the scoring system shown in S1A Fig, gross lesions of the embryonic body were noted and recorded. There were no statistical differences among the three strains (A17-DW-005, A18-MRA-014, and SG9R) when 10^6^ CFU was inoculated into chicken embryos by the AC (*P* > 0.05). When 10^4^ CFU was inoculated, there was no statistical difference between A17-DW-005 and A18-MRA-014 (*P* > 0.05). However, when 10^2^ CFU was inoculated, there was a significant statistical difference among the three strains (*P* < 0.05) (S1B Fig).

Based on the scoring system of S2A Fig, the gross lesions of the embryonic liver were scored. There were no statistical differences among the three strains (A17-DW-005, A18-MRA-014, and SG9R) when 10^6^ and 10^4^ CFU were inoculated, respectively (*P* > 0.05). However, when 10^2^ CFU was inoculated, there was a significant statistical difference among the three strains (*P* < 0.05) (S2B Fig). Hence, when 10^2^ CFU was inoculated into chicken embryos by the AC, virulent, moderately virulent and avirulent strains could also be distinguished based on the scoring results of the gross lesions of the embryonic body and embryonic liver.

### Correlation between growth *in ovo* and virulence

**Fig 1:**
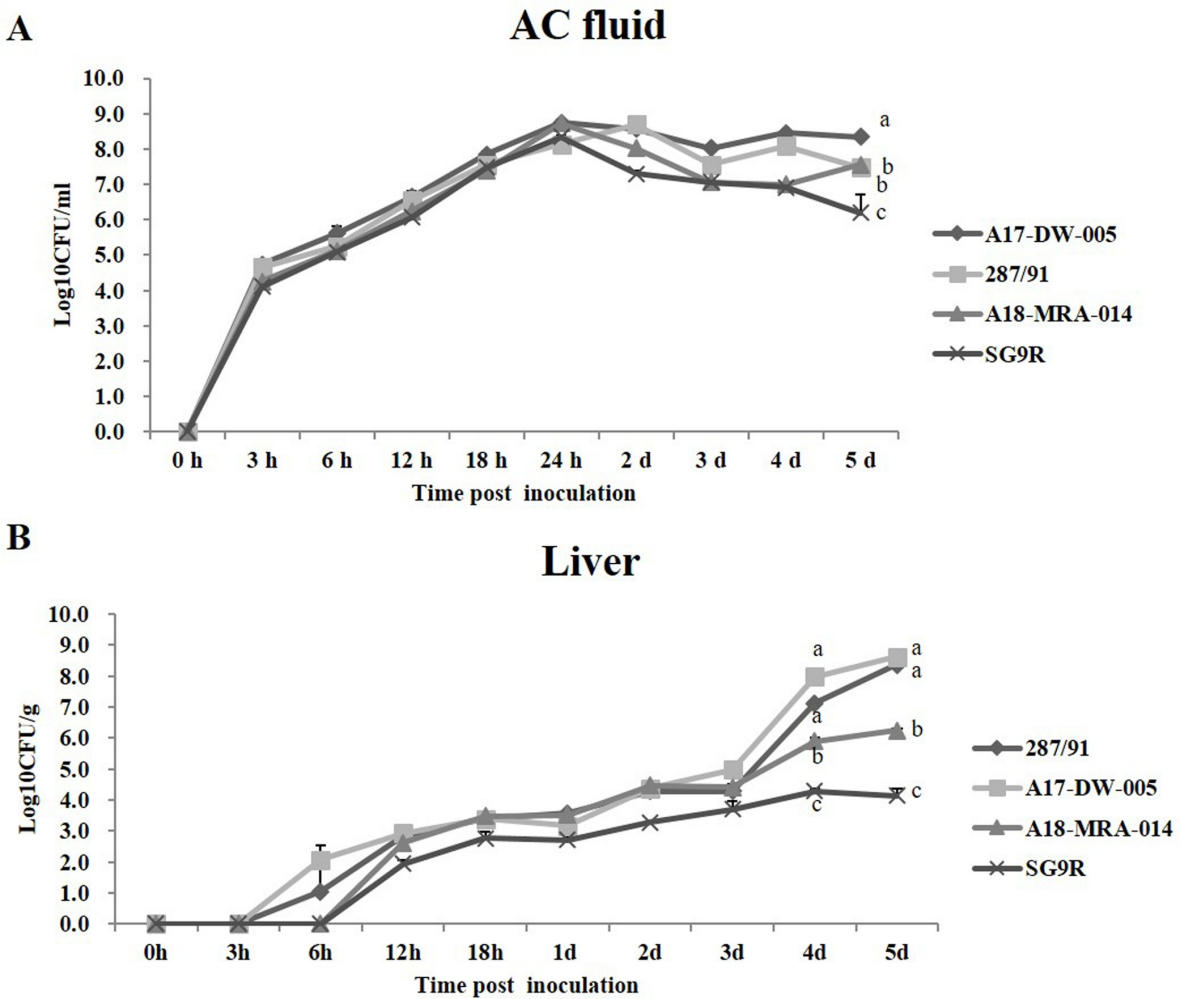
Growth curves of 287/91, A17-DW-005, A18-MRA-014 and SG9R strains after inoculation with 10^4^ CFU into the AC of 13-day-old embryonic chicken eggs (*n* = 3). (A) The mean CFU present in the AC fluid was followed over time. (B) The mean CFU present in the embryonic liver was followed over time. Each point represents the mean ± standard deviation of two embryos per group. None of the strains were recovered from control eggs inoculated with PBS alone.

*In ovo* growth curves were determined in both the AC fluid and the embryonic livers to investigate the correlation between replication (or invasion capability) of *S*. Gallinarum and strain virulence (Fig 1). By comparing the bacterial loads in the AC fluid, it could be observed within the first 24 hours that the four strains multiplied at almost the same rate and reached about 10^8^ CFU/ml. The bacterial counts of the virulent and moderately virulent strains were maintained at 10^7^ to 10^8^ CFU/ml at 2−5 dpi. However, the bacterial count of the avirulent strain decreased slightly at 2 dpi, and was maintained at 10^6^ to 10^7^ CFU/ml up to 5 dpi. By comparing the bacterial counts in the livers, the numbers of virulent, moderately virulent and avirulent strains were significantly different at 4 and 5 dpi (*P* < 0.001). At 5 dpi, the bacterial numbers of the virulent and moderately virulent strains in the liver were about 10,000 and 100 times higher than that of the avirulent strain, respectively.

### Virulence evaluation of field strains by ELA

**Fig 2:**
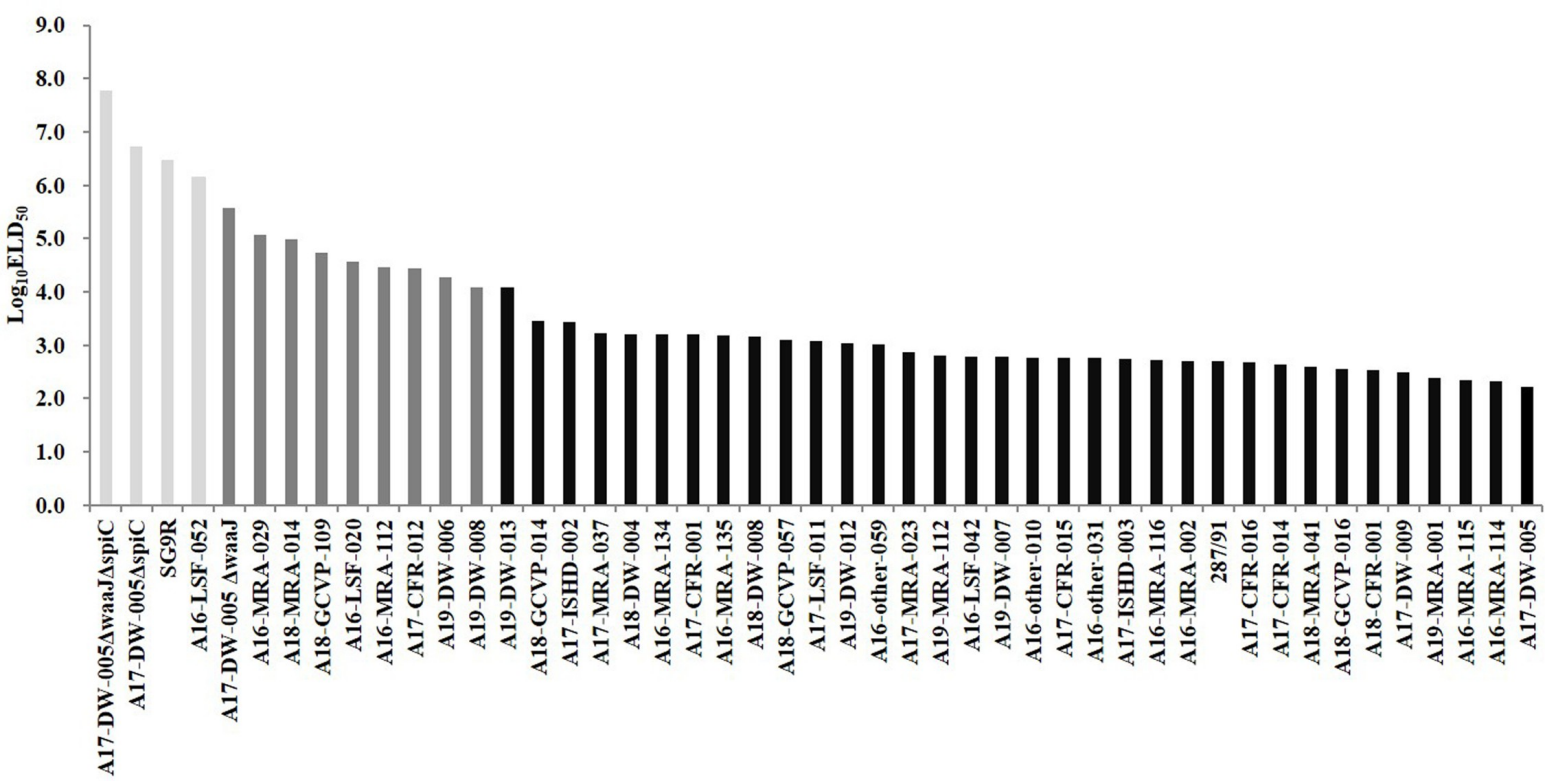
Log_10_ELD_50_ of 13-day-old chicken embryos inoculated by the AC with *S*. Gallinarum field strains in the chicken ELA. The avirulent strains (Log_10_ELD_50_ ≥ 6.1) were displayed in the light grey columns; the moderately virulent strains (4.0 < Log_10_ELD_50_ > 6.1) were displayed in the deep grey columns; the virulent strains (Log_10_ELD_50_ ≤ 4.0) were displayed in the black columns.

Forty-two field strains (shown in Table 3) isolated from clinical cases, including the eight *S*. Gallinarum strains challenged in chickens, were inoculated into 13-day-old embryos via the AC to confirm their virulence (Fig 2). The ELA results of the 42 field strains showed that thirty-two strains (76.2%) were virulent, nine were moderately virulent (21.4%), and one strain was avirulent (2.4%).

**Table 3:** Strains of *S*. Gallinarum used for the ELA in this study.

No.	Isolate	Source	Breed	Age (days)	Year
1	A16-LSF-020	Liver	Native	34	2016
2	A16-LSF-042	Liver	Layer	360	2016
3	A16-LSF-052	Liver	Layer	602	2016
4	A16-MRA-002	Liver	Layer	179	2016
5	A16-MRA-029	Liver	Broiler	25	2016
6	A16-MRA-112	Liver	Broiler	8	2016
7	A16-MRA-114	Liver	Broiler	9	2016
8	A16-MRA-115	Liver	Broiler	7	2016
9	A16-MRA-116	Liver	Broiler	8	2016
10	A16-MRA-134	Liver	Broiler	28	2016
11	A16-MRA-135	Liver	Broiler	9	2016
12	A16-Other-010	Liver	Native	70	2016
13	A16-Other-031	Liver	Layer	245	2016
14	A16-Other-059	Liver	Broiler	9	2016
15	A17-CFR-001	Liver	Broiler	9	2017
16	A17-ISHD-002	Liver	Native	44	2017
17	A17-MRA-023	Liver	Layer	245	2017
18	A17-ISHD-003	Liver	Native	57	2017
19	A17-MRA-037	Liver	Native	95	2017
20	A17-LSF-011	Liver	Native	49	2017
21	A17-CFR-012	Liver	Broiler	18	2017
22	A17-CFR-014	Liver	Broiler	8	2017
23	A17-DW-005	Liver	Broiler	10	2017
24	A17-DW-009	Liver	Broiler	9	2017
25	A17-CFR-015	Liver	Broiler	8	2017
26	A17-CFR-016	Liver	Broiler	7	2017
27	A18-DW-004	Liver	Broiler	10	2018
28	A18-GCVP-014	Liver	Layer	457	2018
29	A18-GCVP-016	Liver	Layer	35	2018
30	A18-MRA-014	Liver	Layer	237	2018
31	A18-GCVP-057	Liver	Layer	60	2018
32	A18-DW-008	Liver	Broiler	8	2018
33	A18-MRA-041	Liver	Broiler	11	2018
34	A18-GCVP-109	Liver	Layer	59	2018
35	A18-CFR-001	Liver	Broiler	10	2019
36	A19-MRA-001	Liver	Broiler	9	2019
37	A19-DW-006	Liver	Broiler	7	2019
38	A19-DW-007	Liver	Broiler	8	2019
39	A19-DW-008	Liver	Broiler	10	2019
40	A19-MRA-112	Liver	Broiler	7	2019
41	A19-DW-012	Liver	Broiler	12	2019
42	A19-DW-013	Liver	Broiler	8	2019

## Discussion

The chicken pathogenicity test is the traditional method used for assessing the virulence of *S*. Gallinarum. However, the use of chicken models is limited by several factors, including ethical considerations, costs, and the requirements for specialized facilities. For decades, embryonic eggs have been used as a convenient alternative infection model for studying viruses and evaluating bacterial virulence [9]. However, to our knowledge, embryos have never been used as a model to study the virulence of *S*. Gallinarum. Studies have shown that for *S*. Pullorum and *S*. Typhimurium, the higher the virulence of the strain in chickens, the higher the mortality rate of the strain in chicken embryos, suggesting that ELA could be used to assess the virulence of *S*. Pullorum and *S*. Typhimurium strains. Hence, in the present study, we analyzed whether chicken embryos could be used as a model for studying the virulence of *S*. Gallinarum.

According to the published literature, both chicken embryo age and inoculation route have a marked effect on the lethality of chicken embryos. The susceptibility of different chicken embryo ages (8 day, 13 day, 16 day) to virulent, partially virulent and avirulent strains of *E*. *coli* has been studied, which showed that 16-day-old chicken embryos were the most resistant [11]. The effect of the embryos age on the susceptibility to lethal infection with *R*. *anatipestifer* was also reported: 7 day > 10 day > 13 day > 15 day [18]. In addition, similar experimental results have been reported for fungal chicken embryo infection tests [8, 30].

Consistent with these studies, our results also showed that with increasing age, the embryo became more resistant (Table 1). Except for the inoculation route of YS, as the embryo age increased, the sensitivity of the embryo decreased, and the strain ELD_50_ increased accordingly. It has been suggested that the increased resistance of older embryos to pathogen infection may reflect the maturation of the embryonic immune system. The enhanced immunity can reduce the burden of bacteria on the host and prevent the spread of pathogens, thereby improving the survival rate of the host.

For the embryo inoculation route, we chose the three most commonly used routes, AC, CAM, and YS. First of all, in terms of the YS route, our results showed that 6-day-old embryos were so susceptible that both virulent and avirulent strains caused 100% mortality. Similar results have been reported for *E*. *coli* and *Enterococcus faecalis* (*E*. *faecalis*). The YS route could not distinguish the virulence of *E*. *coli* isolates because both virulent and non-virulent strains had 100% mortality [12]. The YS inoculation route produced high embryo mortality, which made it difficult to distinguish the virulence of *E*. *faecalis* strains and to estimate the LD_50_ [31]. Thus, inoculation via the YS is not appropriate for use in the ELA to estimate the virulence of *S*. Gallinarum strains.

Regarding the AC and CAM inoculation routes, our results showed that, compared with the CAM route, the AC route was better for distinguishing virulent and avirulent strains. The most significant difference (*P* < 0.000001) (S2 Table) was demonstrated in 13-day-old embryos inoculated via the AC route, although differences could also be seen among other age/route combinations (Table 1) [18]. In conclusion, our experimental results emphasized the importance of the age and route of inoculation for the determination of the virulence of *S*. Gallinarum in the ELA.

A comparison of the growth curves of representative virulent, moderately virulent and avirulent strains in the AC showed that the difference in virulence was not related to the proliferation ability of bacteria in the AC. Because the AC fluid itself supports excellent bacterial growth, the proliferation rates of the strains were almost the same in the first 24 hours, with all of the bacterial counts reaching about 10^8^ CFU/ml (Fig 1). Although the number of bacteria in the avirulent strains had decreased over the next few days, it still maintained a bacterial concentration of about 10^6^ CFU/ml.

However, by comparing the colonization levels of bacteria in the liver, it could be seen that the higher the virulence of the strain, the stronger its invasiveness to the liver. By comparing the bacterial counts in the livers, it could be seen that the higher the virulence of the strain, the higher the level of bacterial colonization. On 4 and 5 dpi, the bacterial load of the virulent strain in the liver was 10^3^−10^4^ times higher than that of the avirulent strain. Also, we observed that about 80−90% of embryos inoculated with virulent strains 287/91 and A17-DW-005 died on 4 and 5 dpi, but embryos inoculated with avirulent strain SG9R did not. Therefore, we speculate that virulent strains have a strong ability to invade the liver, leading to the proliferation of a large number of bacteria, which may be the main reason for embryo death.

Based on the optimal age/route combination of 13 day/AC, we determined the ELD_**50**_ values and 95% CIs of multiple virulent and avirulent representative strains. By comparing the 95% CIs, we have defined the criteria for distinguishing the virulence of *S*. Gallinarum strains in ELA. Regarding the criteria for virulence classification, the selection of representative strains is the first consideration. According to the results of the chicken pathogenicity test, 287/91, A17-DW-005, and A18-GCVP-014 all showed 100% mortality, so they were selected as representative virulent strains (Table 2). In contrast, SG9R, A17-DW-005Δ*spiC*, and A17-DW-005Δ*waaJ*Δ*spiC*, which showed 0% mortality, were selected as representative avirulent strains.

Secondly, how to classify the virulence is the second issue to be considered. In the present study, we clearly observed a correlation between the inoculation dose and embryo mortality. Although no reports on ELD_**50**_ have been found in the *Salmonella*-related literature, ELD_**50**_ has been used to distinguish virulence in other bacteria. It was reported that there was at least a 6-log difference between the least virulent (ELD_**50**_, > 3.3×10^**8**^) and most virulent (ELD_**50**_, 2.2×10^**2**^) *Campylobacter jejuni* strains [17]. In addition, for *Neisseria meningitidis*, it was reported that low virulence strains had an ELD_**50**_ of 10^**3**^ CFU or greater, and high virulence strains had an ELD_**50**_ of approximately 10^**1**^ or less [27]. In our results, there was up to 3.5-log difference between the ELD_**50**_ values of the virulent and avirulent strains, which indicated that the virulence of *S*. Gallinarum strains could be accurately distinguished by ELD_**50**_ (Table 1). In addition, as shown in Table 2, there was a clear correspondence between the chicken pathogenicity test and ELA. The virulence of *S*. Gallinarum on chickens was reflected in the ELA. The results of these studies indicated that ELA could be used as an alternative method to evaluate the virulence of *S*. Gallinarum.

According to the criteria defined in the present study, in the 42 field strains of *S*. Gallinarum, most of the strains (76.2%) isolated were virulent, a small part (21.4%) were moderately virulent, and only one (2.4%) was avirulent. In the present study, most of the strains were virulent or moderately virulent. This may be because the majority of the strains were isolated from sick and dead chickens. In addition, the successful isolation of an avirulent strain indicated that ELA could be used to screen for natural attenuated vaccine candidates. These results verified that the ELA can be applied to evaluate the virulence of field strains of *S*. Gallinarum.

## Supporting information

S1 Table The death pattern of the representative strains. (DOCX)

S2 Table Statistical analysis between ELD_50_ values of virulent and avirulent strains in different age/route combinations. (DOCX)

S1 Fig Scores of gross lesions of the embryonic body. (A) Scoring standard for the chicken embryonic body. (a) A score of 1 for a normal body. (b) A score of 2 for a medium body size. (c) A score of 3 for a small body size. (d) A score of 4 for a small body size plus hemorrhage. (B) Scores of the embryonic body after inoculation with 10^6^, 10^4^, or 10^2^ CFU of A17-DW-005, 18-MRA-014 and SG9R.
(TIF)

S2 Fig Scores of gross lesions of the embryonic liver. (A) Scoring standard for the gross lesions in the liver from chicken embryos. (a) A score of 1 for normal. (b) A score of 2 for swelling. (c) A score of 3 for a few necrotic foci. (d) A score of 4 for many necrotic foci. (B) Scores of embryo liver after inoculation with 10^6^, 10^4^, or 10^2^ CFU of A17-DW-005, 18-MRA-014 and SG9R.
(TIF)
