# Supplementary material for: The use of embryonic chicken eggs as an alternative model to evaluate the virulence of Salmonella enterica serovar Gallinarum
Source: PLoS One. 2020 Sep 10;15(9):e0238630. doi: 10.1371/journal.pone.0238630 (PMC7500061; doi:10.1371/journal.pone.0238630)
Supplement: S1 Table — (DOCX) [file pone.0238630.s001.docx]

**S1 Table. The death pattern of the representative strains.**

| **Strain** | **Chicken embryo mortality (%)** | | | | | | | | |
| --- | --- | --- | --- | --- | --- | --- | --- | --- | --- |
|  | **10^8a^** | **10^7^** | **10^6^** | **10^5^** | **10^4^** | **10^3^** | **10^2^** | **10^1^** |  |
| 287/91 | 100.0 (15/15)^b^ | 100.0  (15/15) | 100.0  (15/15) | 100.0  (15/15) | 93.3  (14/15) | 60.0  (9/15) | 46.7  (7/15) | 20.0  (3/15) |  |
| A17-DW-005 | 100.0  (15/15) | 100.0  (15/15) | 100.0  (15/15) | 100.0  (15/15) | 86.7  (13/15) | 80.0  (12/15) | 53.3  (8/15) | 26.7  (4/15) |  |
| A18-GCVP-014 | 100.0  (15/15) | 100.0  (15/15) | 93.3  (14/15) | 80.0  (15/15) | 66.7  (10/15) | 40.0  (6/15) | 26.7  (4/15) | 0.0  (0/15) |  |
| A18-MRA-014 | 100.0  (15/15) | 100.0  (15/15) | 73.3  (11/15) | 46.7  (7/15) | 40.0  (6/15) | 26.7  (4/15) | 20.0  (3/15) | 0.0  (0/15) |  |
| SG9R | 100.0  (15/15) | 73.3  (11/15) | 46.7  (7/15) | 13.3  (2/15) | 0.0  (0/15) | 0.0  (0/15) | 0.0  (0/15) | 0.0  (0/15) |  |
| A17-DW-005Δ*spiC* | 80.0  (12/15) | 60.0  (9/15) | 40.0  (6/15) | 20.0  (3/15) | 20.0  (3/15) | 0.0  (0/15) | 0.0  (0/15) | 0.0  (0/15) |  |
| 005Δ*waaJ*Δ*spiC* | 66.7  (0/15) | 33.3  (0/15) | 20.0  (0/15) | 6.7  (0/15) | 6.7  (0/15) | 0.0  (0/15) | 0.0  (0/15) | 0.0  (0/15) |  |

^a^95% CI: 95% confidence interval.

^b^Dead embryo/total embryo.
