# Supplementary material for: The use of embryonic chicken eggs as an alternative model to evaluate the virulence of Salmonella enterica serovar Gallinarum
Source: PLoS One. 2020 Sep 10;15(9):e0238630. doi: 10.1371/journal.pone.0238630 (PMC7500061; doi:10.1371/journal.pone.0238630)
Supplement: S2 Table — (DOCX) [file pone.0238630.s002.docx]

**S2 Table. Statistical analysis between ELD_50_ values of virulent and avirulent strains in different age/route combinations.**

| **Virulence** | **Strain** | **Inoculation**  **route^b^** | ***P* value^a^** | |
| --- | --- | --- | --- | --- |
|  |  |  | **13 day^c^** | **16 day** |
| Virulent | 287/91 | AC | 0.000000 | 0.000001 |
|  | A17-DW-005 |  | 0.000000 | 0.000001 |
|  | A18-GCVP-014 |  | 0.000001 | 0.000004 |
| Avirulent | SG9R |  |  |  |
| Virulent | 287/91 | CAM | 0.000063 | 0.000924 |
|  | A17-DW-005 |  | 0.000021 | 0.000700 |
|  | A18-GCVP-014 |  | 0.001371 | 0.006511 |
| Avirulent | SG9R |  |  |  |

^a^The one-way analysis of variance (ANOVA) was used for the analysis of significant differences between ELD_50_ values of virulent and avirulent strains using different age/route combinations. The *P* values were obtained by comparing three virulent strains with the avirulent strain SG9R respectively.

^b^Chicken embryos were inoculated via Allantoic cavity (AC) and chorioallantoic membrane (CAM).

^c^13-day-old and 16-day-old embryos were used for in the present study.
