# Supplementary figures and images for: The use of embryonic chicken eggs as an alternative model to evaluate the virulence of Salmonella enterica serovar Gallinarum
Source: PLoS One. 2020 Sep 10;15(9):e0238630. doi: 10.1371/journal.pone.0238630 (PMC7500061; doi:10.1371/journal.pone.0238630)

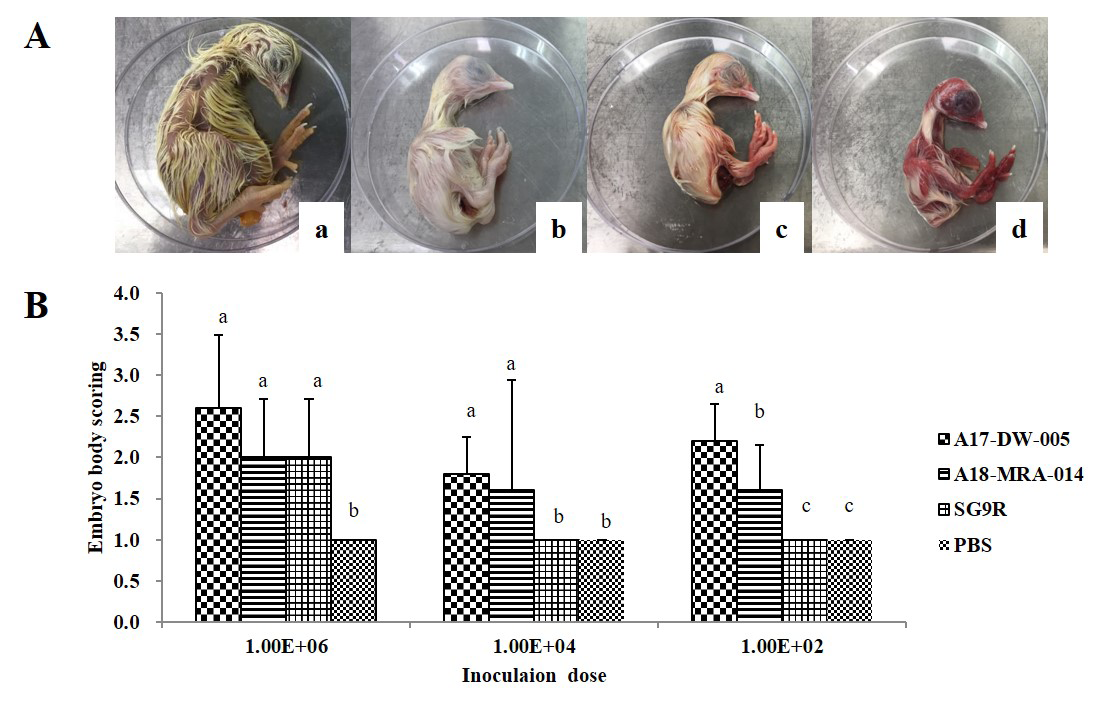

Supplement: S1 Fig — (A) Scoring standard for the chicken embryonic body. (a) A score of 1 for a normal body. (b) A score of 2 for a medium body size. (c) A score of 3 for a small body size. (d) A score of 4 for a small body size plus hemorrhage. (B) Scores of the embryonic body after inoculation with 106, 104, or 102 CFU of A17-DW-005, 18-MRA-014 and SG9R. (TIF) [file pone.0238630.s003.tif]

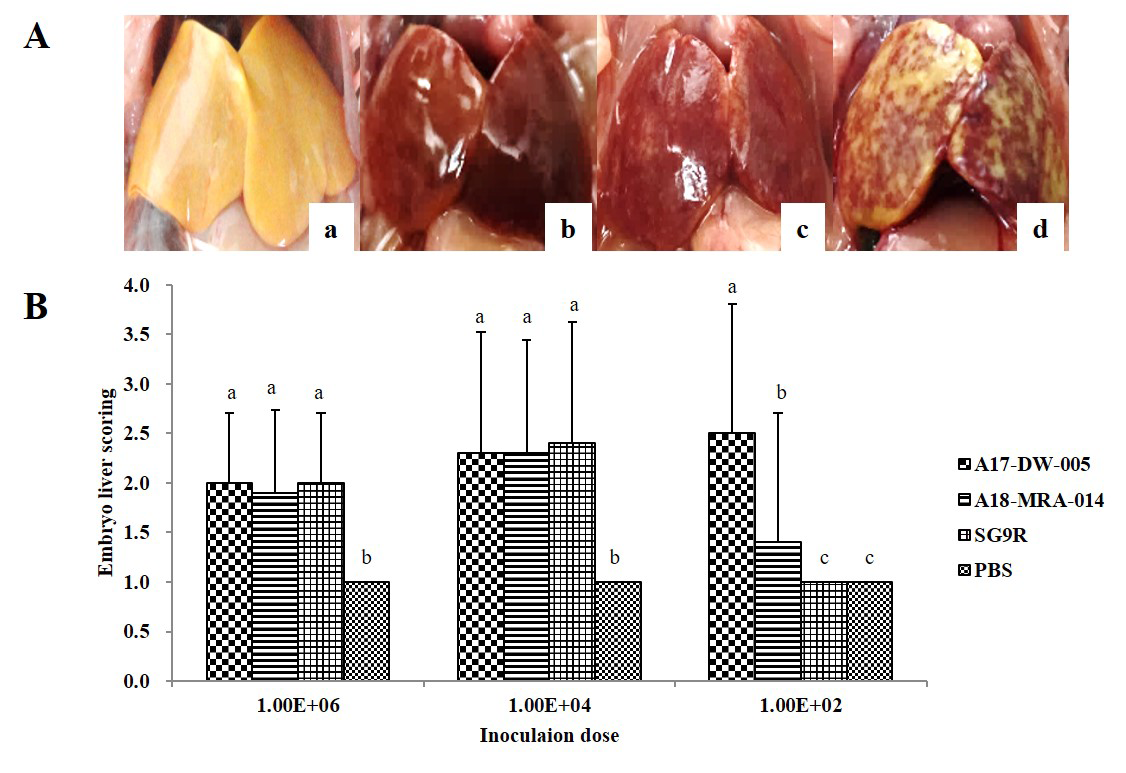

Supplement: S2 Fig — (A) Scoring standard for the gross lesions in the liver from chicken embryos. (a) A score of 1 for normal. (b) A score of 2 for swelling. (c) A score of 3 for a few necrotic foci. (d) A score of 4 for many necrotic foci. (B) Scores of embryo liver after inoculation with 106, 104, or 102 CFU of A17-DW-005, 18-MRA-014 and SG9R. (TIF) [file pone.0238630.s004.tif]
